# Enhanced gene transfection efficiency by low-dose 25 kDa polyethylenimine by the assistance of 1.8 kDa polyethylenimine

**DOI:** 10.1080/10717544.2018.1510065

**Published:** 2018-09-21

**Authors:** Hui Zhang, Zhiyi Chen, Meng Du, Yue Li, Yuhao Chen

**Affiliations:** Laboratory of Ultrasound Molecular Imaging, Department of Ultrasound Medicine, The Third Affiliated Hospital of Guangzhou Medical University, Guangzhou, China

**Keywords:** Non-viral vectors, gene delivery, polyethylenimine, gene therapy

## Abstract

Gene therapy is a promising strategy for treatments of various diseases. Efficient and safe introduction of therapeutic genes into targeted cells is essential to realize functions of the genes. High-molecular-weight polyethylenimines (HMW PEIs) including 25 kDa branched PEI and 22 kDa linear PEI are widely used for *in vitro* gene transfection. However, high-gene transfection efficiency is usually accompanied with high cytotoxicity, which hampers their further clinical study. On the contrary, low-molecular-weight polyethylenimines (LMW PEIs) such as 1.8 kDa PEI and 800 Da PEI show good biocompatibility but their applications are limited by the poor DNA condensation capability. In this study, we find that 1.8 kDa PEI, but not 800 Da PEI combined with low-dose 25 kDa PEI could significantly promote gene transfection with low cytotoxicity. Plasmids encoding enhanced green fluorescence protein (EGFP) were delivered by the combined PEI and gene transfection efficiency was evaluated by microscopic observation and flow cytometry. Parameters including concentrations of 25 kDa PEI and 1.8 kDa PEI and preparation ways were further optimized. This study presents an efficient and safe combined PEI-based non-viral gene delivery strategy with potential for *in vivo* applications.

## Introduction

Gene therapy shows great promise in the treatment of diseases such as inherited disorders, cardiovascular diseases, and cancers (Chen et al., 2013; Naldini, 2015; Lino et al., 2018). However, the central issue that needs to be solved is to safely and efficiently transfer genes of interest into targeted cells through layers of barriers such as membrane, endosome, lysozyme, and nuclear membrane (Chen et al., 2013; Ying et al., 2016). With the development of material science, non-viral vectors are intensively explored to deliver therapeutic DNAs or RNAs *in vitro* or *in vivo*. Compared with viral vectors, non-viral vectors have the advantages of low immunogenicity and cytotoxicity, ease of modification with targeting ligand and powerful gene-loading capacity without size limitation. In recent years, non-viral vectors based on liposomes, polymers, and nanoparticles are hotspots (Nayerossadat et al., 2012; Li et al., 2016; Shen et al., 2016; Zhang et al., 2018).

Since first reported in 1995, PEI has been becoming one of the most widely studied gene vehicles characterized with high positive charge density and ‘proton sponge effect’, by which genes can be robustly condensed and protected from endosome or lysozyme capture (Boussif et al., 1995; Regnström et al., 2003). Transfection reagents such as Gen500, jetPEI, which is developed based on HMW PEIs, are commercially available (Li et al., 2015; Cao et al., 2018). However, high-gene transfection efficiency always comes along with high cytotoxicity by HMW PEIs (Chollet et al., 2002). Further applications of HMW PEIs in gene therapy are hindered by their cytotoxicity, especially when administrated at high doses. Although low-dose PEIs do not show obvious toxicity, they fail to efficiently deliver genes into cells, especially for those hard-to-transfect cell lines. Even by deliberately designing HMW PEI-based transfection agents, HMW PEIs still have the potential for gene therapy. Extensive endeavors have been made to lower adverse effects of HMW PEIs in the meanwhile preserving the transfection efficiency. Common strategies include decorating the backbone of HMW PEIs to lower the strong positive charge, which causes interactions with cell membrane and cytotoxicity (Mao et al., 2006; Zheng et al., 2016), or combining with other methods (Chen et al., 2012).

LMW PEIs have drawn much attention for their low cytotoxicity. For example, LMW PEI cross-linked by disulfide bonds could efficiently condense nucleic acids and can be degraded in a redox-responsive manner in cytoplasm (Taranejoo et al., 2015), which has been one of the most successful approaches for the application of LMW PEI as a gene vehicle. In recent years, LMW PEI has been extensively exploited to realize high-gene transfection efficiency with minimal cytotoxicity (Son et al., 2012). LMW PEIs are commonly combined with other materials such as liposome (Hanzlíková et al., 2009) and chitosan (Gao et al., 2010) or modified with certain ligands (Zhou et al., 2014; Hu et al., 2016). Modifications with ligands such as lipoic (Meng et al., 2011) and acid zinc(II) coordinative module (Shuai et al., 2017) can further facilitate DNA condensation, hence promoting gene delivery. However, although various modifications to some extent solve the dilemma between gene transfection and cytotoxicity, the complication of material modification or synthesis and time- and cost- inefficiency may restrict their universal applications.

It has been proposed that in an optimized HMW PEI/DNA transfection system, only a small portion of PEI could condense DNA to form PEI/DNA polyplexes while most of the PEI exists in a free state. The free PEI substantially enhances gene transfection but is also the leading cause of cytotoxicity. It is presumed that the free PEI could promote endosome escape during which genes could be released into the cytoplasm and enter in nuclei (Boeckle et al., 2004). Cai et al. confirmed this point and extensively illustrated the mechanism of how free cationic polymer promotes gene transfection in subcellular and molecular levels (Cai et al., 2016). Based on these findings, it is reasonable to replace the free HMW PEI by low-toxic agents to enhance gene transfection efficiency and lower cytotoxicity. In the present study, we utilize LMW PEI with a molecular weight of 1.8 kDa or 800 Da to promote gene delivery by HMW PEI/DNA polyplexes and develop a safe and efficiency combined PEI-based gene delivery strategy.

## Materials and methods

### Materials

DMEM basic medium, OPTI-MEM medium, calf serum, trypsin and penicillin-streptomycin solution (100 U/ml) were from Gibco (Carlsbad, CA, USA). Ovarian cancer cell line A2780 and breast cancer cell line MDA-MB-231 were from ATCC (Manassas, VA). Cell counting kit (CCK-8) was purchased from Dojindo (Kumamoto, Japan). The plasmid encoding EGFP (pGFP) is kept in our own laboratory. 25 kDa, 1.8 kDa and 800 Da branched PEIs were purchased from Sigma-Aldrich (Madrid, Spain). Lipofectamine 2000 and PureLink^TM^ HiPure Plasmid Miniprep Kit were purchased from Invitrogen (Carlsbad, USA). Reagents for gel electrophoresis including 1 kb DNA ladder, 6 × loading buffer (15% ficoll), 4S Red Plus and agarose were from Sangon Biotech (Shanghai, China).

### Cell culture

Both A2780 cells and MDA-MB-231 cells were cultured in DMEM medium which was supplemented with 10% calf serum and 1% antibiotics. All cells were grown in an incubator at 37 °C with 5% CO_2_. The culture was changed into fresh medium every 24 h. Cells were passaged when the confluence of the cells is up to about 90%. The culture was exchanged into DMEM medium containing 10% calf serum without antibiotics the day before gene transfection.

### Preparation of PEI transfection agent and DNA binding analysis

PEI with a molecular weight of 25 kDa, 1.8 kDa, or 800 Da was accurately weighed and dissolved into distilled water to reach a concentration of 10 μg/ml, respectively. The solution was filtered by 0.2 μm filters and stored in 4 °C. When used, the stock PEI was properly diluted 10 times or 100 times by distilled water. PEI 25 kDa was mixed with DNA to reach a certain N/P ratio and the complexes were incubated at room temperature for 10 min. N/P ratio was calculated referring to previously reported (Wan et al., 2015).

The DNA binding ability of 25 kDa PEI was evaluated by agarose gel electrophoresis. The DNA/PEI polyplexes were loaded into 0.7% agarose gel. Electrophoresis was run in TAE buffer at 120 V for 20 min. The gel was subsequently stained by DNA dyes and analyzed under an electrophoresis gel imaging system.

### Gene transfection *in vitro*

A2780 cells were inoculated at a density of 1 × 10^5^ cells/ml into 2.5 ml antibiotic-free DMEM medium supplemented with 10% calf serum in a 6-well plate the other night. For MDA-MB-231 cells, which grow more slowly, an inoculating density of 6 × 10^5^ cells/ml was applied. Before transfection, the medium was exchanged into 2 ml serum-free OPTI-MEM medium. A portion of PEI (25 kDa, 1.8 kDa or 800 kDa) was mixed with 1 μg plasmid DNA and incubated for 10 min. For combined PEI transfection, an aliquot of 25 kDa PEI was firstly mixed with 1 μg plasmid DNA and the mixture was incubated for 10 min (25 kDa PEI +1 μg pGFP). Subsequently, a certain amount of 1.8 kDa PEI or 800 Da PEI was added into the complexes and incubated for another 10 min (25 kDa PEI +1 μg pGFP +1.8 kDa PEI or 800 Da PEI). OPTI-MEM medium (500 μl) was added into the mixture. Subsequently, the solution was transferred into the cell culture. After 4 h incubation in a 37 °C, 5% CO_2_ incubator, the medium was exchanged into fresh antibiotics-free DMEM medium containing 10% calf serum. After 24 h, cells expressing EGFP were analyzed under a fluorescence microscope.

In addition, another way to prepare the combined PEI/DNA solution was adopted to evaluate the role of 1.8 kDa PEI. Namely, 1.8 kDa PEI was first incubated with pGFP for 10 min. Then 25 kDa PEI was added and incubated for 10 min (50 μg 1.8 kDa PEI +1 μg pGFP +0.5 μg 25 kDa PEI).

### Cytotoxicity analysis

CCK-8 reagent was used to evaluate the cell viability after transfection. The cytotoxicity of 25 kDa PEI/DNA with different N/P ratios was analyzed at a cell confluence of about 70%. A2780 cells were seeded into a 96-well plate and incubated for 24 h. Next day, cells were treated as above-mentioned PEI transfection protocol. On the third day, the medium in each well was exchanged into 100 μl fresh DMEM medium supplemented with 10% calf serum and 10% 10 μl CCK-8 reagent. The culture was further incubated in a 37 °C, 5% CO_2_ incubator for 2 h. The absorbance at 450 nm was analyzed by a microplate reader (Bio-Tek ELx808, Winooski, VT, USA). Each experiment was performed independently three times. Cell viability (%) was calculated in accordance to the following equation: $\text{Cell viability }\left(\%\right)=\left(A_{\text{sample}}-A_{\text{blank}}\right)/(A_{\text{control}}-A_{\text{blank}})$.

In addition, the cytotoxicity of the combined PEI/DNA mixture and lipofectamine 2000/DNA complexes was evaluated and compared at various cell confluences. A2780 cells of 2 × 10^4^ cells/well, 1 × 10^4^ cells/well, 5 × 10^3^ cells/well and 1 × 10^3^ cells/well were seeded into a 96-well plate to reach various cell confluences.

### Evaluation of gene transfection efficiency

EGFP expression was evaluated under an inverted fluorescence microscope (OLYMPUS CKX41, Japan). Subsequently, flow cytometry was utilized to quantify gene transfection efficiency. Briefly, cells were washed by PBS twice, digested by trypsin and harvested by centrifugation (300 × g, 5 min). The cells were washed twice by PBS and suspended in PBS at a density of 1 × 10^6^ cells/ml. The percentage of EGFP-positive cells was measured by flow cytometry (BD Accuri C6, San Jose, CA, USA). Each experiment was performed independently three times.

### Statistical analysis

GraphPad Prism 5.0 software (San Diego, CA, USA) was utilized to perform statistical analysis. All the data were expressed as the mean ± SD. All the experiments were performed independently three times. Group comparisons were performed by Student’s *t*-test. *p* < .05 means that there is statistically significant difference between two groups.

## Results and discussion

### DNA binding ability of 25 kDa PEI analysis

**Figure 1.**
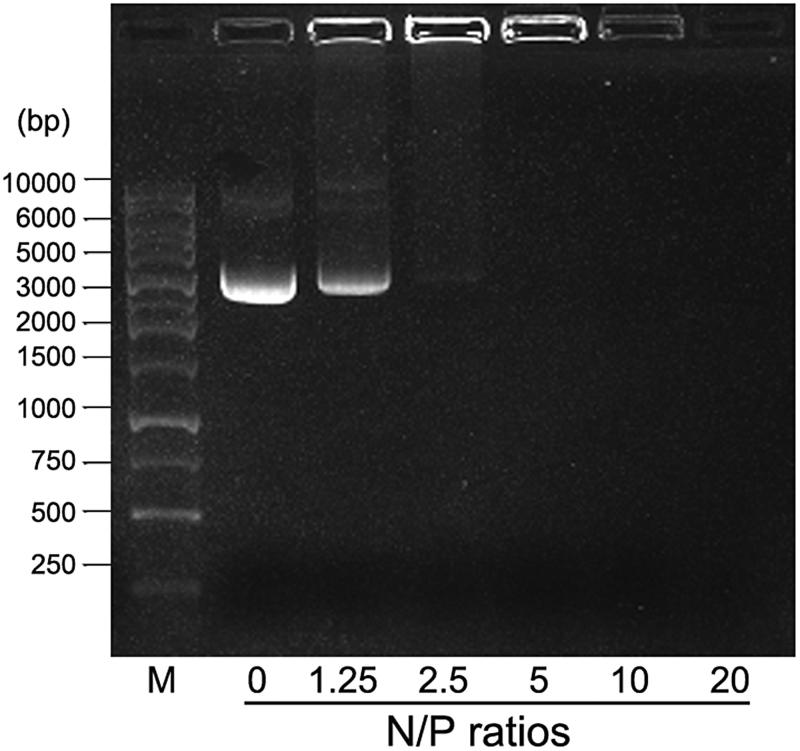
DNA binding analysis of 25 kDa PEI by gel retardation assay. M: DNA marker; the values 0, 1.25, 2.5, 5, 10, and 20 denote different N/P ratios.

It is demonstrated that HMW PEI with high-positive charge is powerful to condense nucleic acids into compact DNA/PEI nanoparticles with sizes ranging from 100 nm to 500 nm under different N/P ratios (Alipour et al., 2017). As shown in Figure 1, DNA condensation ability of 25 kDa PEI improves with increasing N/P ratio (weight ratios consistent with N/P ratios are as follows: N/*P* = 0, 1 μg pGFP; N/*P* = 1.25, 1 μg pGFP +0.16 μg PEI; N/*P* = 2.5, 1 μg pGFP +0.32 μg PEI; N/*P* = 5, 1 μg pGFP +0.64 μg PEI; N/*P* = 10, 1 μg pGFP +1.28 μg PEI; N/*P* = 20, 1 μg pGFP +2.56 μg PEI). When the N/P ratio is above 5, no free DNA could be observed on the gel, indicating that the PEI is excessive and free PEI exists. At a point between N/P ratio 2.5 and N/P ratio 5, DNA is completely condensed with no free DNA and PEI left in the solution. In this study, we define that when N/P ratio is 4, pure DNA/PEI polyplexes with no or limited free DNA and PEI could be achieved, by which the formula of the mixture is 1 μg pGFP +0.5 μg 25 kDa PEI.

### Gene delivery by pure 25 kDa PEI/DNA complexes combined with LMW PEI

**Figure 2.**
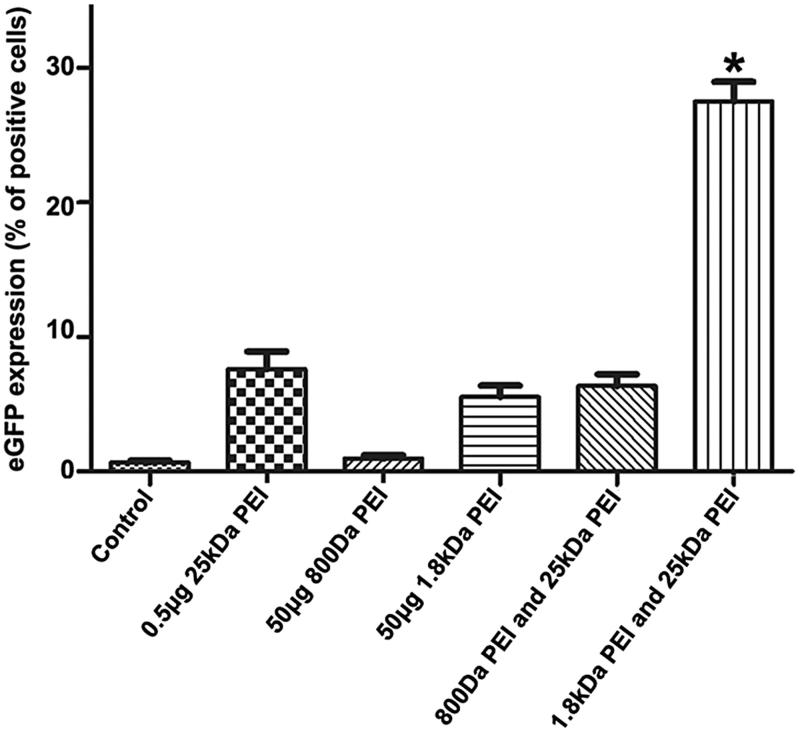
Evaluation of transfection efficiency by the combination of 25 kDa PEI and LMW PEI (800 Da PEI or 1.8 kDa PEI). From left to right, cells without any treatment is used as the negative control, cells are transfected by 0.5 μg 25 kDa PEI +1 μg pGFP, 50 μg 800 Da PEI +1 μg pGFP, 50 μg 1.8 kDa PEI +1 μg pGFP, 0.5 μg 25 kDa PEI +1 μg pGFP +50 μg 800 Da PEI and 0.5 μg 25 kDa PEI +1 μg pGFP +50 μg 1.8 kDa PEI, respectively. Gene transfection efficiency of each group is quantified by flow cytometry. Each column represents the mean ± S.D. (*n* = 3). *p < .05 versus other groups.

In our study, LMW PEIs including 1.8 kDa PEI and 800 kDa PEI are employed to enhance *in vitro* gene delivery by 25 kDa PEI. Unless otherwise stated, the cell line used as a model is A2780. All the fluorescence microscopic images in this study are shown in Supplementary materials. As a proof-of-concept experiment, low-dose 25 kDa PEI (0.5 μg) is incubated with 1 μg pGFP (N/*P* = 4) to form pure polyplexes. Subsequently, 50 μg 1.8 kDa PEI or 800 Da PEI was added into the polyplexes. As shown in Figure 2, when N/P ratio of 25 kDa PEI is 4 (0.5 μg 25 kDa PEI), gene transfection efficiency is about 8%, indicating that pure 25 kDa PEI/DNA polyplexes have limited gene transfer ability. From another point of view, the free 25 kDa PEI plays an essential role in DNA transportation into cell nuclei. Although LMW PEIs show good biocompatibility, gene delivery capability by 800 Da or 1.8 kDa PEI is poor with transfection efficiency of appropriate 2% (50 μg 800 Da PEI) and 5% (50 μg 1.8 kDa PEI), respectively, even though a high concentration is adopted. LWM PEIs are added into the 25 kDa PEI/DNA complexes to ask whether they can serve as substitutes for free 25 kDa PEI to facilitate gene transfection. Intriguingly, 1.8 kDa PEI, but not 800 Da PEI (0.5 μg 25 kDa PEI +50 μg 800 Da PEI), could significantly improve gene delivery. The transfection efficiency by the minimal dose of 25 kDa PEI by the assistance of 1.8 kDa PEI (0.5 μg 25 kDa PEI +50 μg 1.8 kDa PEI) is significantly enhanced compared with other groups (about 30%). It has been reported that PEI with a molecular weight as low as 600 Da could promote gene transfer by a cationic cholesterol-based nanoparticle vector by dissociating DNA from the complex and releasing pDNA from endosome to cytoplasm by the proton sponge effect (Hattori Maitani, 2007). In our case, PEI with a molecular weight of 800 Da has no favorable effects on pure 25 kDa PEI/DNA complexes transportation, while the mechanism is still to be elucidated.

### Effects of combined PEI/DNA preparation ways on gene transfection

The combined PEI-based gene delivery strategy presented in our study is built up upon the conclusion that free cationic chains play an imperative role in PEI/DNA complex transportation by promoting endosome escape. In the process of PEI/DNA reagent preparation, it is reasonable to first incubate 25 kDa PEI and DNA for a while to form compact nanoparticles, subsequently mix 1.8 kDa PEI into the solution, which could exist in a free state. On the other hand, if 1.8 kDa PEI is mixed with DNA first, the polyplexes formed with loose structure will compete with the post-added 25 kDa PEI for DNA binding, and theoretically, the gene transfection efficiency would be impaired. The preparation of the transfection reagent in the order of 25 kDa PEI, pGFP and 1.8 kDa PEI resulted in the highest transfection efficiency (about 30%), while for the group that LMW and pGFP are mixed prior to HMW, the gene transfection efficiency decreases obviously to about 7% (Figure S2).

### Optimization of N\P ratio of 25 kDa PEI and pGFP

**Figure 3.**
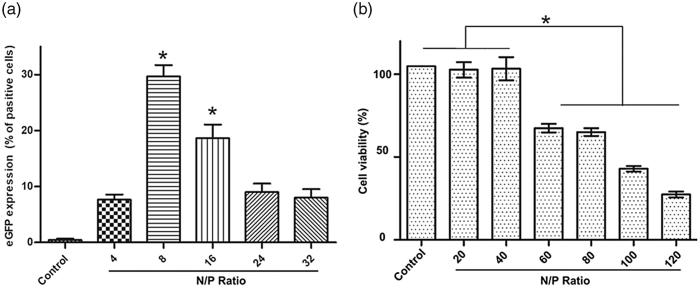
pGFP introduction into A2780 cells by 25 kDa PEI at different N/P ratio. (a) Gene transfection efficiency quantified by flow cytometry (mean ± SD, *n* = 3, *p < .05); (b) Cytotoxicity of 25 kDa PEI/DNA complexes against A2780 cells at various concentrations by CCK-8 assay (mean ± SD, *n* = 3, *p < .05).

N/P ratio plays an essential role in gene delivery by affecting the characteristics of the PEI/DNA polyplexes including size and charge. As shown in Figure 3(a), the percentage of cells expressing EGFP transfected by 25 kDa PEI is the highest at the N/P ratio of 8 and is in obvious decline when the N/P ratio is above 8, although the morphology of A2780 cells treated with PEI/DNA at higher N/P ratios (8, 16, 24, 32) does not change obviously under bright field. In addition, CCK-8 assay reveals that PEI/DNA with a N/P value up to 40 shows no obvious toxicity to the cells. For PEI/DNA with a N/P ratio between 20 and 40, EGFP expression is not shown to be consistent with cytotoxicity. Cell viability decreases dramatically when the N/P ratio is above 40 (Figure 3(b)). Apoptosis induced by the excessive 25 kDa PEI (Hunter, 2006) or other potential affects, which is called phase II cytotoxicity (induced after 24 h incubation) activated by “a mitochondrially mediated apoptotic program” (Moghimi et al., 2005) may be accountable for the decreased transfection efficiency at the N/P ratios ranging from 20 to 40. The cytotoxicity of 1.8 kDa PEI is also investigated and 1.8 kDa PEI does not show obvious toxicity even at a high concentration (over 20 μg/ml) (data not shown).

### Optimization of the working concentration of 1.8 kDa PEI

**Figure 4.**
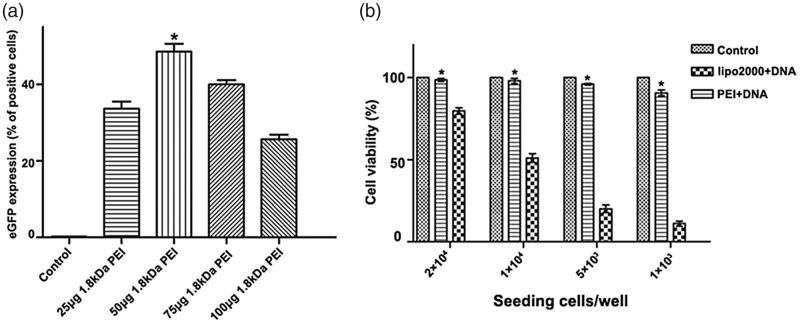
Optimization of 1.8 kDa PEI working concentration. (a) A2780 cells are transfected by (from left to right) control, 1 μg 25 kDa PEI +1 μg pGFP +25 μg 1.8 kDa PEI, 1 μg 25 kDa PEI +1 μg pGFP +50 μg 1.8 kDa PEI, 1 μg 25 kDa PEI +1 μg pGFP +75 μg 1.8 kDa PEI and 1 μg 25 kDa PEI +1 μg pGFP +100 μg 1.8 kDa PEI, respectively. Gene transfection efficiency for each group is quantified by flow cytometry. (mean ± SD, *n* = 3, *p < .05 versus control, 25 μg 1.8 kDa pEI, 75 μg 1.8 kDa PEI and 100 μg 1.8 kDa PEI groups) (g). (b) Analysis and comparison of the cytotoxicity of the combined PEI and lipofectamine 2000 under various cell confluences. Different amount of A2780 cells are inoculated in a 96-well plate to acquire various cell confluences. (mean ± SD, *n* = 3, *p < .05).

Since the innate cytotoxicity of HMW PEI at a higher concentration (N/*P* > 8) lowers gene delivery efficiency, we need to know whether PEI-based gene transfection efficiency could be elevated to a new level. Based on the optimal N/P ratio of 8 for 25 kDa PEI, involvement of 1.8 kDa PEI is investigated and its working concentration is further optimized. The amount of 25 kDa PEI and plasmid DNA is kept at N/P ratio of 8, by which 1 μg 25 kDa PEI and 1 μg pGFP are included and incubated at room temperature for 10 min. Subsequently, various amounts of 1.8 kDa PEI (25 μg, 50 μg, 75 μg and 100 μg) is mixed into the solution with further 10 min incubation, respectively. As shown in Figure 4(a), gene transfection efficiency by a dose of 25 μg 1.8 kDa PEI combined with 25 kDa PEI is only about 35%. When 1.8 kDa up to 50 μg is adopted, gene transfection efficiency increases significantly to about 48%. It has been reported that low dose of free LMW PEIs have limited facilitating effects on polyplexes delivery due to less efficacious endosome-lysosome pathway blocking [23], but the authors have not investigated higher doses of LMW PEI. Through optimization, the recommended formula of the combined PEI reagent for A2780 cells is: 1 μg 25 kDa PEI +1 μg pGPF +50 μg 1.8 kDa PEI.

Cytotoxicity of the combined PEI is further evaluated and compared with lipofectamine 2000. Some transfection reagents show cell damage in a cell density-dependent way. For instance, for lipofectamine 2000, to minimize the toxicity, transfection experiment should be performed when cells grow to a confluence of above 90%, referring to the manufacturer’s instruction. This is also confirmed in our study that cell viability losses in negative correlation with cell confluence for lipofectamine 2000. For the combined PEI/DNA agent presented in this study, no obvious cytotoxicity is observed even at a low cell confluence when only 1 × 10^3^ cell/well was seeded the day before (Figure 4(b)). As to the aspect of biocompatibility, the combined PEI is superior to lipofectamine 2000.

**Figure 5.**
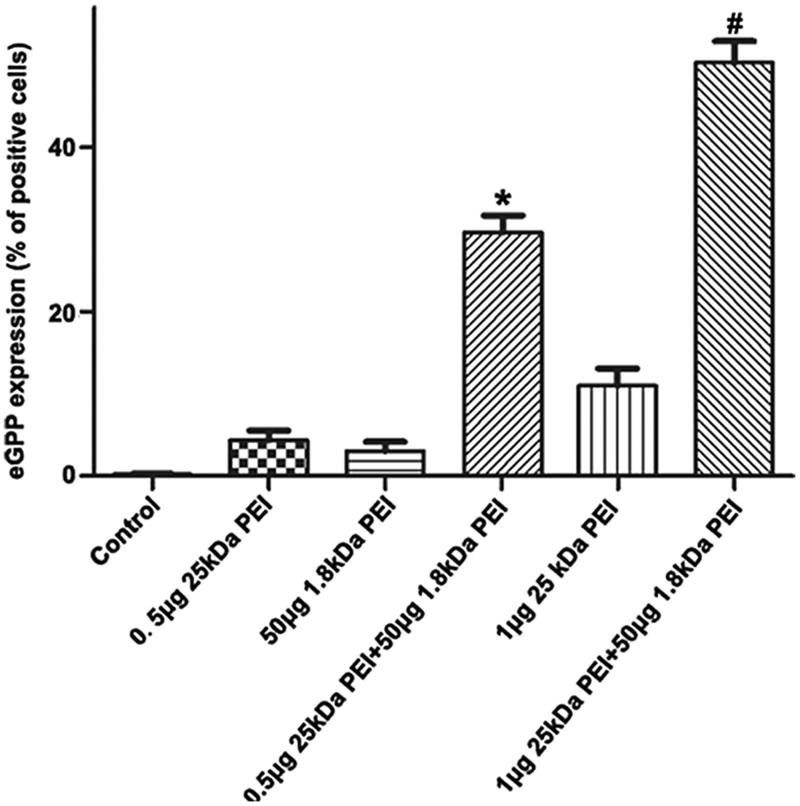
Analysis of gene transfection efficiency in MDA-MB-231 cells by combined PEI. The adherent cells are transfected by negative control, 0.5 μg 25 kDa PEI +1 μg pGFP, 50 μg 1.8 kDa PEI +1 μg pGFP, 0.5 μg 25 kDa PEI +1 μg pGFP +50 μg 1.8 kDa PEI, 1 μg 25 kDa PEI +1 μg pGFP and 1 μg 25 kDa PEI +1 μg pGFP +50 μg 1.8 kDa PEI. Flow cytometry is utilized to quantify the corresponding efficiency. (mean ± SD, *n* = 3, *p < .05 versus control, 0.5 μg 25 kDa PEI and 50 μg 1.8 kDa PEI groups, #*p* < .05 versus control, 1 μg 25 kDa PEI and 50 μg 1.8 kDa PEI groups).

The breast cancer cell line MDA-MB-231 is utilized in our study to further justify the combined PEI strategy (Figure 5). For MDA-MB-231 cells, gene transfer by sole 25 kDa PEI (N/*P* = 4) or 50 μg 1.8 kDa PEI results in only about 5% and 3% gene transfection efficiency, respectively. Even the N/P ratio of 25 kDa PEI and pGFP is up to 8, there is a limited increase in the efficiency (about 10%). By combination of 25 kDa PEI and 1.8 kDa PEI, gene delivery is significantly promoted with the efficiency of about 30% for (0.5 μg 25 kDa PEI +1 μg pGFP +50 μg 1.8 kDa PEI) group and about 55% for (1 μg 25 kDa PEI +1 μg pGFP +50 μg 1.8 kDa PEI) group. Since MDA-MB-231 cells grow much more slowly than A2780 cells, the EGFP-expressing cells in the fluorescence images is sparser than that of A2780 cells due to the low cell confluences (Figure S5). It is noteworthy that the parameters utilized is obtained from A2780 cell line. For MDA-MB-231 or other cells lines, to get the best results, the system may need pertinent adjustments. Certainly, to confirm the applicability of this combined PEI transfection system, more cell lines including those harder-to-transfect cells will be tested.

## Conclusions

In this study, we replace free 25 kDa PEI with 1.8 kDa PEI and ask whether low toxic LMW could promote gene transfection efficiency and in the meanwhile lower cytotoxicity. Without complicated synthesis or modification procedure, low-dose 25 kDa PEI, DNA and 1.8 kDa PEI are simply mixed in a sequential order. Interestingly, the gene transfection efficiency increases significantly and no obvious cytotoxicity is observed. The underlying mechanism is tentatively verified by gene transfection by the combined PEI and DNA reagent through different preparation ways. It is imperative to incubate 25 kDa PEI and DNA first for maturation, then the 1.8 kDa PEI is included. Addition of the 1.8 kDa prior to 25 kDa PEI results in poor gene transfection efficiency. The combined PEI system does not show obvious cytotoxicity within the experimental ranges tested here, indicating that it is a safe gene delivery tool. A2780 cell line and MDA-MB-231 cell line are involved in the study and more cell lines will be used to validate this non-viral system.

In conclusion, this study presents a low-toxic, simple, low-cost, and efficient gene transfection strategy based on combined PEI with potential for *in vivo* gene therapy studies.

## Supplementary Material

supplementary_material.docx
