# Supplementary material for: Enhanced gene transfection efficiency by low-dose 25 kDa polyethylenimine by the assistance of 1.8 kDa polyethylenimine
Source: Drug Deliv. 2018 Sep 21;25(1):1740–5. doi: 10.1080/10717544.2018.1510065 (PMC6161618; doi:10.1080/10717544.2018.1510065)
Supplement: supplementary_material.docx [file IDRD_A_1510065_SM4686.docx]

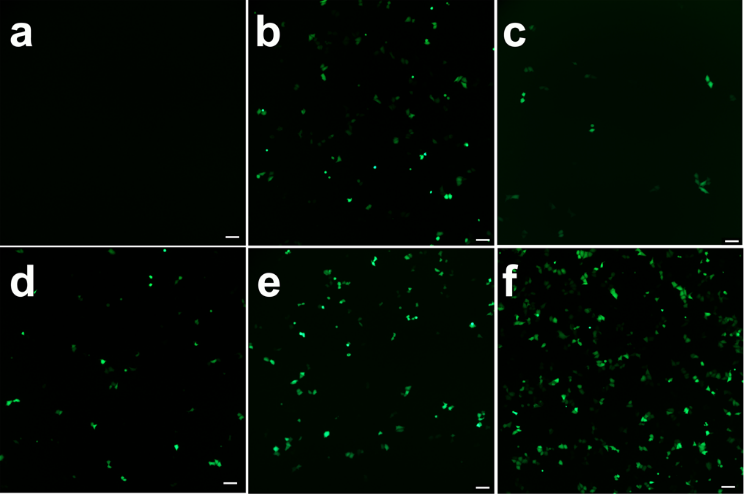


Figure S1. Evaluation of transfection efficiency by the combination of­ 25 kDa PEI and LMW PEI (800 Da PEI or 1.8 kDa PEI). Fluorescence microscope (a-f) was utilized to analyze the transfection efficiency. a: cells without any treatment as negative control; b: cells transfected by 0.5 μg 25 kDa PEI+1μg pGFP; c: 50 μg 800 Da PEI+1μg pGFP; d: 50 μg 1.8 kDa PEI + 1μg pGFP; e: 0.5 μg 25 kDa PEI + 1μg pGFP+50 μg 800 Da PEI; f: 0.5 μg 25 kDa PEI + 1 μg pGFP + 50 μg 1.8 kDa PEI. Note: Scale bar = 10 μm.


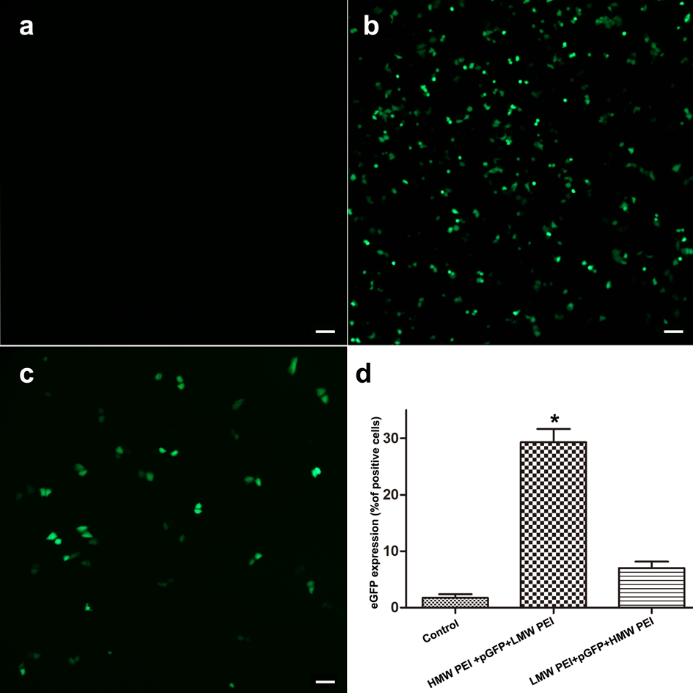


Figure S2. Transfection by combined PEI prepared in different ways. a: control; The transfection reagent is prepared in an order of 25 kDa PEI, pGFP and 1.8 kDa PEI (b) or in an order of 1.8 kDa PEI, pGFP and 25 kDa PEI (c). Note: Scale bar = 10 μm; Gene transfection efficiency quantified by flow cytometry (d). Each column represents the mean ± S.D. (n = 3). ^*^P < 0.05 versus other groups.


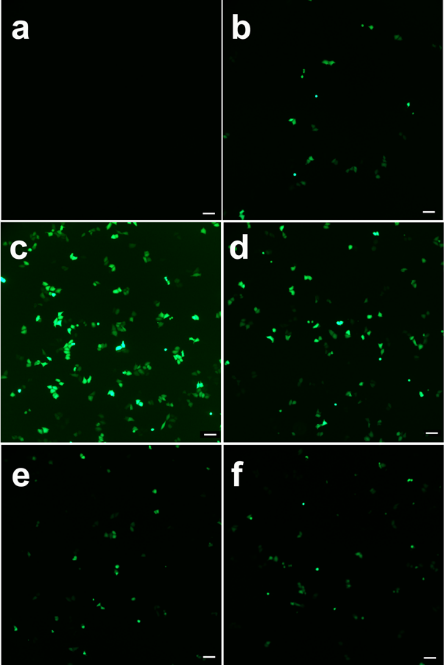


Figure S3. pGFP introduction into A2780 cells by 25 kDa PEI at different N/P ratio. a: control; b: N/P = 4, c: N/P = 8, d: N/P =16, e: N/P = 24, f: N/P = 32. The amount of plasmid DNA is kept 1 μg in all groups in a six-well plate. Note: Scale bar = 10 μm.


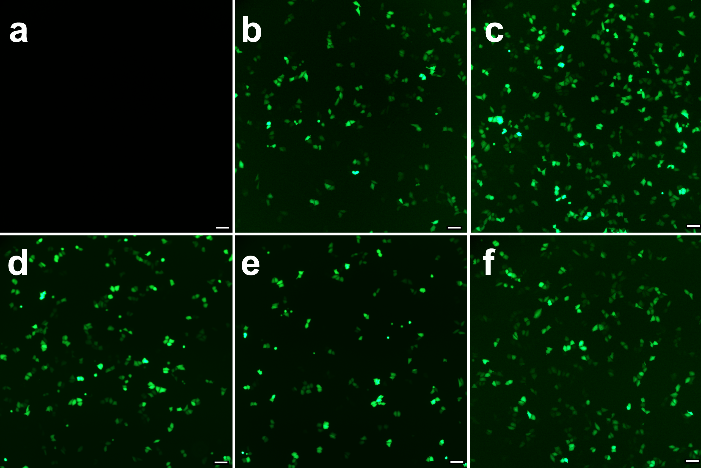


Figure S4. Optimization of 1.8 kDa PEI working concentration. A2780 cells are transfected by control (a), 1 μg 25 kDa PEI + 1 μg pGFP + 25 μg 1.8 kDa PEI (b), 1 μg 25 kDa PEI + 1 μg pGFP + 50 μg 1.8 kDa PEI (c), 1 μg 25 kDa PEI + 1 μg pGFP + 75 μg 1.8 kDa PEI (d), 1 μg 25 kDa PEI + 1 μg pGFP + 100 μg 1.8 kDa PEI (e) and 10 μl lipofectamine2000 + 4 μg pGFP (f), respectively. Note: Scale bar = 10 μm.


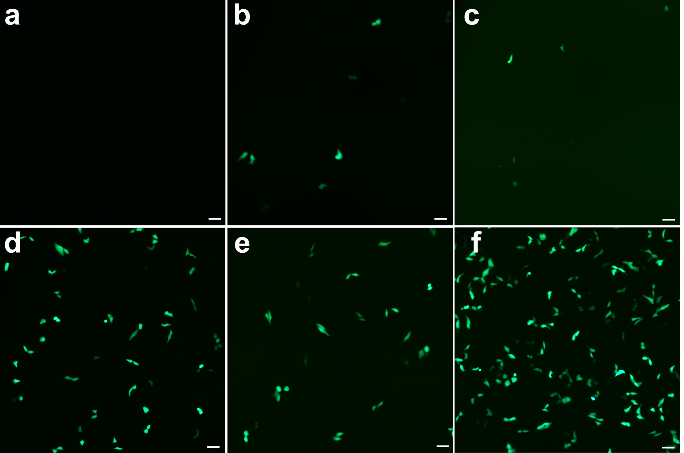


Figure S5. Analysis of gene transfection efficiency in MDA-MB-231 cells by combined PEI. a: negative control; the adherent cells are transfected by 0.5 μg 25 kDa PEI + 1 μg pGFP (b), 50 μg 1.8 kDa PEI + 1 μg pGFP (c), 0.5 μg 25 kDa PEI + 1 μg pGFP + 50 μg 1.8 kDa PEI (d); 1 μg 25 kDa PEI + 1 μg pGFP (e); 1 μg 25 kDa PEI + 1 μg pGFP + 50 μg 1.8 kDa PEI (f). Note: Scale bar = 10 μm.
